# Safety evaluation of remdesivir administration in patients with severe renal impairment and coronavirus disease: a systematic review and meta-analysis

**DOI:** 10.1186/s12879-025-11153-5

**Published:** 2025-06-02

**Authors:** Takumi Umemura, Hideo Kato, Yoshikazu Mutoh, Mao Hagihara, Yoshiaki Ikeda, Hiroshige Mikamo

**Affiliations:** 1 Department of Clinical Infectious Diseases, Aichi Medical University, Nagakute, Aichi 480-1195 Japan; 2 Department of Clinical Infectious Diseases, Tosei General Hospital, Seto, Aichi Japan; 3 Department of Pharmacy, Tosei General Hospital, Seto, Aichi Japan; 4 College of Pharmacy, Kinjo Gakuin University, Nagoya, Aichi Japan; 5 Department of Pharmacy, Mie University Hospital, Tsu, Mie Japan

**Keywords:** Remdesivir, Renal impairment, SARS-CoV-2

## Abstract

**Background:**

We conducted a comprehensive systematic review and meta-analysis to evaluate whether remdesivir (RDV) is safe for patients with severe renal impairment (SRI) and COVID-19, compared to non-SRI patients or those not receiving RDV.

**Methods:**

This study was conducted according to the PRISMA guidelines for reporting systematic reviews and meta-analyses. We searched PubMed, Cohcrane, CINAHL, and Ichushi databases up to October 11, 2024. The outcomes assessed kidney injury, hepatic disorder and mortality. Randomized controlled trials and retrospective and cohort studies reporting kidney injury, hepatotoxicity, and mortality in (i) SRI patients treated with RDV versus without RDV or (ii) SRI patients versus non-SRI patients treated with RDV were included. Targeted patients were defined as adults with COVID-19 based on a positive reverse transcription polymerase chain reaction or rapid antigen test for SARS-CoV-2 from nasopharyngeal or salivary swabs regardless of symptoms.

**Results:**

One randomized controlled trial and 14 cohort studies met the inclusion criteria and were included in the final meta-analysis. Among SRI patients, RDV significantly reduced the incidence of kidney injury (risk ratio [RR] = 0.51, 95% confidence interval [CI] = 0.27–0.97) but had no significant difference in the development of hepatic disorder (RR = 0.88, 95% CI = 0.39–1.98) and mortality (RR = 0.79, 95% CI = 0.55–1.15). In the comparison between SRI and non-SRI patients treated with RDV, SRI patients demonstrated a significantly higher incidence of kidney injury (odds ratio [OR] = 2.51, 95% CI = 1.49–4.23), with no significant difference in the development of hepatic disorder (OR = 1.04, 95% CI = 0.43–2.53). Meanwhile, SRI patients treated with RDV exhibited significantly higher mortality than non-SRI patients treated with RDV (OR = 2.20, 95% CI = 1.51–3.22).

**Conclusion:**

Our meta-analysis demonstrated that RDV administration in SRI patients with COVID-19 was safe compared to non-SRI or SRI patient treated without RDV. We suggest that the use of RDV should be actively considered for SRI patients.

**Supplementary Information:**

The online version contains supplementary material available at 10.1186/s12879-025-11153-5.

## Background

Remdesivir (RDV) is an adenosine analog that undergoes intracellular metabolism to form an active triphosphorylated form. The active form is subsequently incorporated into the nascent viral RNA chain of severe acute respiratory syndrome coronavirus 2 (SARS-CoV-2), where it acts as a chain terminator, thereby disrupting viral replication and inactivating the virus at an early stage [1, 2]. Because of this mechanism of action, RDV has been a critical therapeutic option for the management of coronavirus disease (COVID-19). Clinical studies have demonstrated its efficacy in treating moderate-to-severe cases of COVID-19 as well as its ability to prevent progression to severe disease in high-risk patients. These findings have led to recommendations supporting the use of RDV in mild-to-severe COVID-19 cases [3, 4]. However, the safety profile of RDV, particularly in vulnerable populations including those with varying degrees of renal impairment is uncertain. The development of renal and hepatic toxicities is one of the most notable adverse events [5]. Pharmacokinetic studies have demonstrated that exposure to RDV and its metabolites (GS-441524 and GS-704277) is not significantly influenced by renal function or the timing of RDV administration during dialysis sessions [6]. Real-world data have provided insight into the use of RDV in patients with severe renal impairment (SRI). Yamada et al. conducted a nationwide cohort study and reported improved mortality outcomes associated with the use of RDV in patients with SRI, highlighting the potential benefits of RDV for this vulnerable population [7]. However, safety-related concerns have been raised in some real-world studies, although these studies are often limited by small sample sizes and inconsistencies in comparison groups, making it difficult to draw definitive conclusions [8, 9]. Furthermore, studies offering an integrated evaluation of the available data, particularly regarding the balance between the efficacy and safety of RDV in patients with SRIs and COVID-19, are lacking. Thus, we conducted a comprehensive systematic review and meta-analysis to evaluate the safety of RDV administration in patients with SRI compared to that in patients without SRI or in those not receiving RDV.

## Methods

### Data sources and search strategy

This study was conducted according to the PRISMA guidelines for reporting systematic reviews and meta-analyses (PRISMA checklist) [10]. No protocol was registered for this study. We systematically searched relevant articles written in the English and Japanese languages in the PubMed, Cochrane, CINAHL, and Ichushi databases up to October 11, 2024. The search terms were as follows: “remdesivir” AND “chronic kidney disease” OR “renal failure” OR “renal impairment” OR “end stage renal disease” OR “end-stage renal disease” OR “dialysis” OR “kidney failure” OR “kidney insufficiency” OR “kidney dysfunction” OR “renal insufficiency” OR “renal dysfunction” OR “CKD” OR “ESRD” OR “ESKD” AND “covid 19” OR “covid 19 vaccines” OR “covid 19 serotherapy” OR “covid 19 nucleic acid testing” OR “covid 19 serological testing” OR “covid 19 testing” OR “sars cov 2” OR “severe acute respiratory syndrome coronavirus 2” OR “ncov” OR “coronavirus” OR “cov” OR “sars cov 2” OR “2019 ncov.” The following two PICO criteria were used for study selection: (i) patient population (P), patients with COVID-19 with SRI; intervention (I), RDV treatment; comparison (C), non-antiviral treatment; outcome (O), kidney injury, hepatotoxicity, and mortality; and (ii) (P), patients with COVID-19 with RDV treatment; (I), patients with SIR; (C), patients without SIR; (O), kidney injury, hepatoxicity and mortality.

### Study selection

Titles and abstracts were independently screened by two individuals (TU and HK) to exclude irrelevant articles. Full-text articles were then reviewed based on the inclusion and exclusion criteria, and articles for the final qualitative synthesis and meta-analysis were identified. Any disagreements were resolved through arbitration by a third investigator (MH). If the original publication did not include sufficient information regarding the outcomes, additional data were requested from the corresponding authors via e-mail. Studies were excluded if the authors could not provide such data upon request. Randomized controlled trials (RCTs) and retrospective and cohort studies (CSs) reporting kidney injury, hepatotoxicity, and mortality in (i) patients with SIR treated with RDV or not treated with SRI or (ii) patients with SRI or not treated with RDV were included. Targeted patients were defined as adults with COVID-19 based on a positive reverse transcription polymerase chain reaction or rapid antigen test for SARS-CoV-2 from nasopharyngeal or salivary swabs regardless of symptoms.

### Data extraction and quality assessment

Relevant data extracted from the studies included the authors, publication year, study design and period, drug regimens for each antimicrobial agent, clinical response, treatment duration, demographic features (age), and patient population. The Cochrane Collaboration tool for clinical trials was used to assess the risk of bias in each included RCTs [11]. The following indicators were rated as “low risk,” “high risk,” or “unclear”: randomization sequence generation, allocation concealment, blinding of participants and personnel, blinding of outcome assessments, incomplete outcome data, selective reporting, and inclusion of intention-to treat analyses. The risk of bias was assessed using the risk of bias in non-randomized studies version 2 (ROBINS-I V2). Studies were classified as having a low, medium, serious, or critical risk of bias based on the score of retrospective observational studies [12]. All differences in opinion were resolved by consensus.

### Definitions of safety

The time to follow-up was based on the definition used in each study. The intention-to-treat population was defined as all randomized patients in the RCTs and the safety-evaluable population that had been administered RDV in the SRI setting according to the study protocol and had undergone an assessment of the clinical response. Primary outcomes were kidney injury and hepatotoxicity. The secondary outcome was overall mortality. Kidney injury was evaluated based on serum creatinine levels or meeting the criteria including serum creatinine before treatment. Hepatic disorder was defined by a liver function test (LFT) including aspartate aminotransferase (AST) or alanine transaminase (ALT) elevation above the normal upper limit.

### Statistical analysis

Based on a previous study [13], a meta-analysis was conducted using Review Manager (RevMan Web Version 7.12.0. The Cochrane Collaboration, 2022. Available from: RevMan Cochrane.org). Statistical heterogeneity between studies was evaluated by a χ^2^ test. *I*^2^ was used to denote the degree of heterogeneity (0–25%, low heterogeneity; 25–50%, moderate heterogeneity; 50–75%, substantial heterogeneity; and 75–100%, considerable heterogeneity). Significant heterogeneity was defined as a *p* < 0.1 or *I*^2^ > 50%. Fixed- and random-effects models were applied when data were considered homogeneous or heterogeneous, respectively. If heterogeneity was observed, sensitivity was analyzed. We then performed a sensitivity analysis restricting the low-risk-of-bias studies. Publication bias was assessed using Egger’s test if at least ten studies were classified in this category. Unadjusted risk ratios (RRs), odds ratios (ORs), and 95% confidence intervals (CIs) were calculated for each study. The pooled RRs or ORs and 95% CIs were calculated using a random-effects model (DerSimonian and Laird method), and the RRs or ORs from these results were compared.

## Results

### Systematic review

**Fig. 1:**
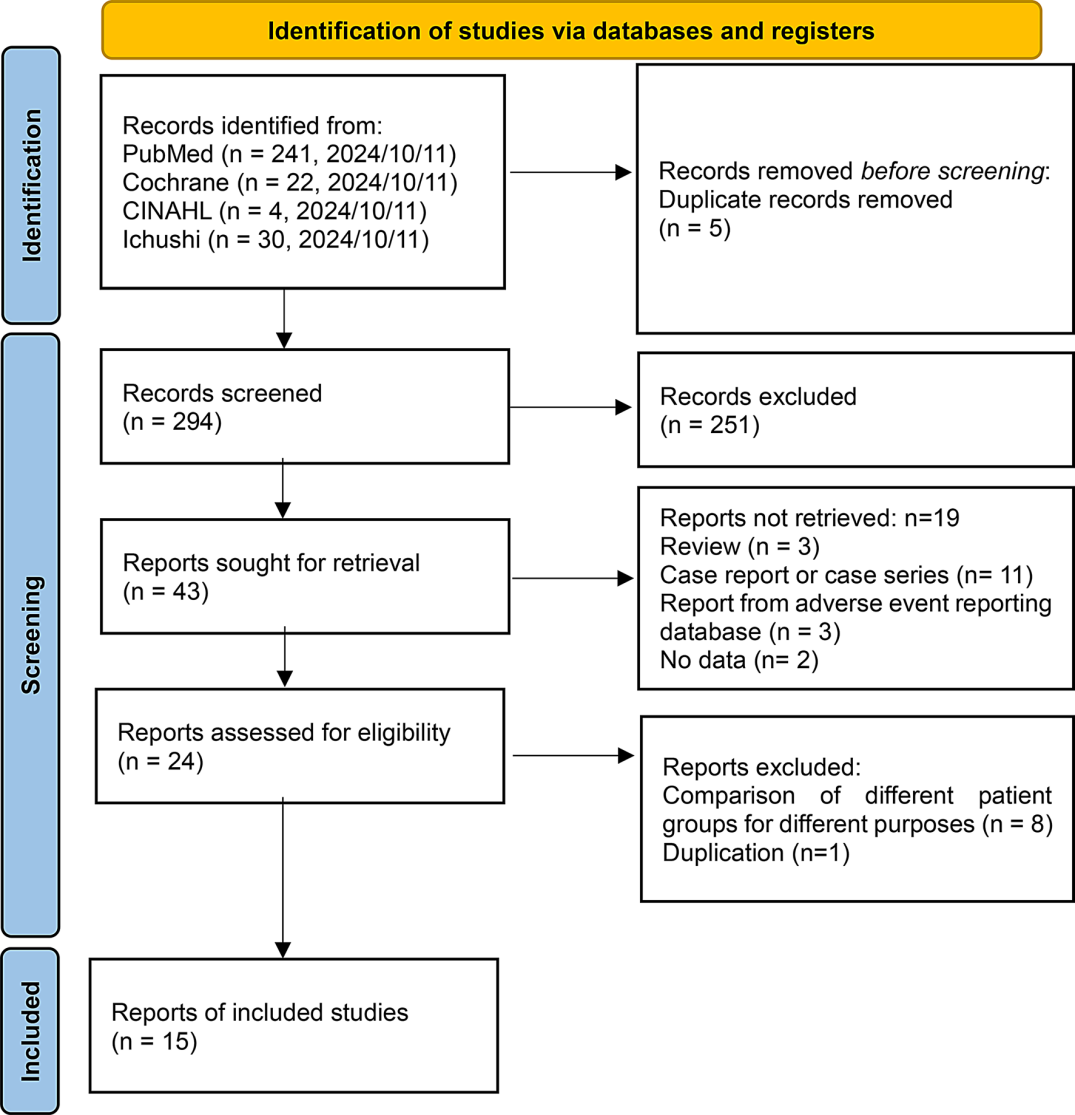
Preferred Reporting Items for Systematic Reviews and Meta-analyses (PRISMA) flow diagram for study selection. This figure shows a PRISMA flowchart. The PubMed, Chocrane, CINAHL, and Ichushi databases were searched for eligible articles using relevant keywords until October 11, 2024

Figure 1 illustrates the study selection process. A total of 297 publications were retrieved. After removing duplicates, 294 publications remained. The titles and abstracts were screened to exclude irrelevant studies, which resulted in 43 potentially eligible studies. Eventually, 1 RCT and 14 CSs fulfilled the inclusion criteria and were included in the final meta-analysis. The characteristics of these studies are summarized in Table 1. The included studies were conducted in the USA [9, 14–16, 18–20], Japan [7, 8, 17], Korea [21, 22], and Taipei [23], and one RCT was conducted in five countries: Brazil, Portugal, Spain, the United Kingdom, and the United States [24]. The Cochrane Collaboration “risk of bias” (ROB) tool was used to assess the quality of the RCT. The included study was registered with the clinical trial registration numbers (EudraCT 2020-005416-22; Clinical Trials.gov NCT04745351). The study by Sise [24] had a low risk (Supplementary Fig. 1). The ROB was assessed in all CSs. Four of 13 CSs used propensity score matching [8, 9, 14, 21], one applied multivariate adjustment [23], and the remaining studies did not adjust for confounding factors. The grading of individual categories and corresponding reasons are provided in Supplementary Table 1 in the supplemental material. In the comparison between patients with SRI treated with RDV and those not treated with RDV, three studies were rated as having low ROB, two studies as having moderate ROB, and one study as having serious ROB. Additionally, in the comparison between patients with SRI treated with RDV and those without SRI, two studies were rated as having a low ROB, three studies had a moderate ROB, and two studies had a serious ROB.

**Table 1:** Characteristics of studies included in the meta-analysis

Author	Study design period	Setting of comparison (RDV with SRI vs. RDV without SRI)	Definition of SRI	Including patients receiving RRT	No. of patients (RDV with SRI vs. comparison)	Treatment duration of RDV (days)	Age (years)	Definition of kidney injury	Definition of hepatic disorder	Mortality
Ackley et al. (2021) [14]	MC, RC May 2020 to Oct 2020	RDV without SRI	eCrCl < 30 mL/min	No	40 vs. 40 by PSM	5 (median)	80 vs. 84 (median)	1.5 times SCr	ALT of ≥ 5 times the ULN	30-day
Schieber et al. (2021) [15]	MC, RC May 2020 to Jul 2020	RDV without SRI	eGFR < 30 mL/min	Yes	21 vs. 130	5 (median)	62 in all patients (mean)	Unknown	LFT of ≥ 5 times the ULN	-
Pettit et al. (2021) [16]	SC, RC May 2020 to Oct 2020	RDV without SRI	eGFR < 30 mL/min	Yes	20 vs. 115	5 (median)	70 vs. 54 (mean)	1.5 times SCr	LFT of ≥ 5 times the ULN	In-hospital
Seethapathy et al. (2022) [9]	MC, RC Mar 2020 to Apr 2020	Non-RDV with SRI	eGFR < 30 mL/min/1.73m^2^	No	31 vs. 31 by PSM	Unknown	41 vs. 36 (mean)	1.5 times SCr	AST or ALT of ≥ 5 times the ULN	28-day
Koga et al. (2022) [17]	SC, RC May 2021 to Apr 2022	Non-RDV with SRI	HD	Yes	35 vs. 15	3–10	64 vs. 57 (median)	–	unknown	-
Sunny S et al. (2022) [18]	SC, RC May 2020 to Apr 2021	RDV without SRI	eCrCl < 30 mL/min	No	55 vs. 361	3–	74 vs. 61 (mean)	AKIN criteria [25]	AST or ALT of ≥ 5 times the ULN	At discharge
Umemura et al. (2023) [8]	SC, RC Mar 2020 to Apr 2022	RDV without SRI	eGFR < 30 mL/min	No	23 vs. 23 by PSM	5 (median)	80 vs. 81 (median)	1.5 times SCr	AST or ALT of ≥ 5 times the ULN	30-day
Zaki et al. (2023) [19]	MC, RC Mar 2020 to Jan 2021	Non-RDV with SRI	HD or PD	Yes	112 vs. 374	4 (median)	65 vs. 54 (mean)	–	AST or ALT of ≥ 5 times the ULN	30-day
Gonzalez et al. (2024) [20]	MC, RC Nov 2020 to Nov 2021	RDV without SRI	eGFR < 30 mL/min/1.73m^2^	Yes	273 vs. 2751	5	67 in all patients (median)	–	–	28-day
Yang et al. (2024) [21]	MC, RC Mar 2020 to Sep 2022	Non-RDV with SRI	eGFR < 30 mL/min/1.73m^2^	Yes	178 vs. 178 by PSM	3–5	75 vs. 74 (median)	new dialysis	-	In-hospital
Park et al. (2024) [22]	MC, RC Nov 2020 to Mar 2022	Non-RDV with SRI	eGFR < 30 mL/min/1.73m^2^	Yes	64 vs. 37	3–5	62 vs. 68 (median)	RIFLE criteria [27]	DILIN criteria [28]	30-day
Chang et al. (2024) [23]	MC, SC Apr 2022 to Oct 2022	RDV without SRI	eGFR < 30 mL/min/1.73m^2^	Yes	307 vs. 1036	3–5	79 vs. 81 (median)	0.3 mg/dL increase or 1.5 times SCr	–	In-hospital
Sise et al. (2024) [24]	MC, RCT Mar 2021 to Mar 2022	Non-RDV with SRI	eGFR < 30 mL/min/1.73m^2^	Yes	163 vs. 80	2–5	68 vs. 71 (mean)	MEDRA [26]	MEDRA [26]	29-day
Yamada et al. (2024) [7]	MC, RC Nov 2020 to Mar 2022	Non-RDV with SRI	Scr > 3 mg/dL or KTR	Yes	272 vs. 1177	Unknown	75 vs. 75 (median)	-	-	28-day
MC, multicenter; SC, single center; RC, retrospective cohort; RCT, randomized control trial; RDV, remdesivir; SRI, severe renal impairment; Scr, serum creatinine; KTR, kidney transplantation; RRT, renal replacement therapy; eCrCl, estimated creatinine clearance; eGFR, estimated glomerular filtration rate; PSM, propensity score matching; AKIN, acute kidney injury network; MEDRA, medical dictionary for regulatory activities; RIFLE, risk/injury/failure/loss end-stage; ALT, alanine aminotransferase; ULN, upper limit of normal; LFT, liver function test; AST, aspartate transaminase; DILIN, drug-induced liver injury network										

Data from 7,942 patients across all studies were extracted. The numbers of SRI patients and non-SRI patients treated with RDV were 855 and 189, respectively. Similarly, 739 SRI patients were treated with RDV and 4,456 patients had no SRI. In 9 of the 13 reports, RDV was administered according to the package insert (200 mg on the first day, followed by 100 mg from the second day onward) [8, 17–24], whereas the dosing regimens in the remaining reports were unknown. Ten of the 14 reports included patients receiving renal replacement therapy (RRT), such as hemodialysis or peritoneal dialysis [8, 16–18, 20–25], and two studies exclusively focused on RRT patients [17, 19]. Second, four CSs used propensity score matching to adjust for patient background in their analyses [8, 9, 14, 21]. Although an Egger’s test was planned, these analyses were not performed because of the limited number of studies eligible for inclusion in the meta-analysis.

## Meta-analysis

### Kidney injury

**Fig. 2:**
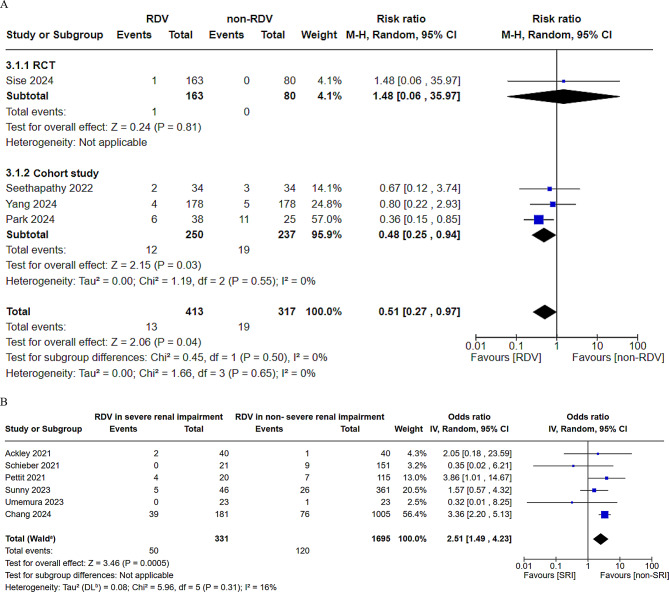
Forest plot of the risk or odds ratio for kidney injury between patients with severe renal impairment. This figure presents a meta-analysis of kidney injuries in patients with severe renal impairment. (**A**) Patients with severe renal impairment (SRI) treated with remdesivir (RDV) vs. those not treated with RDV. (**B**) Patients with SRI treated with RDV versus patients without SRI. RDV, remdesivir; SRI, severe renal impairment; CI, confidence interval

Kidney injury data were extracted from ten CSs and one RCT [8, 9, 14–16, 18, 21–24] and analyzed in two comparisons: between SRI patients treated with and without RDV [9, 17, 21, 22, 24] and between SRI patients and non-SRI patients with RDV [8, 14–16, 18, 23]. In the comparison between SRI patients treated with and without RDV, RDV administration significantly reduced the development of kidney injury (relative risk RR = 0.51, 95% confidence interval CI = 0.27–0.97, heterogeneity *p* = 0.65, Fig. 2A). The incidence of renal injury was significantly lower in non-SRI patients treated with RDV than SRI patients treated with RDV (odds ratio OR = 2.51, 95% CI = 1.49–4.23, heterogeneity *p* = 0.31, Fig. 2B).

### Hepatic disorder

**Fig. 3:**
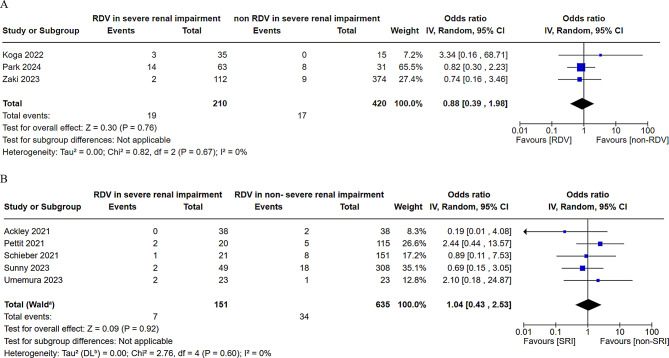
Forest plot of the risk or odds ratio for hepatic disorder between patients with severe renal impairment (SRI).This figure presents the results of a meta-analysis of hepatotoxicity in patients with SRI. (**A**) Patients with SRI treated with remdesivir (RDV) vs. those not treated with RDV. (**B**) Patients with SRI treated with RDV versus patients without SRI. RDV, remdesivir; SRI, severe renal impairment; CI, confidence interval

Hepatic disorder data were extracted from nine CSs [8, 9, 14–19] and analyzed in two comparisons: between SRI patients treated with RDV and those not treated with RDV [17, 19, 22], and between SRI patients treated with RDV and non-SRI patients treated with RDV [8, 14–16, 18]. No significant difference in the development of hepatic disorder was observed between SRI patients treated with RDV and those not treated with RDV (RR = 0.88, 95% CI = 0.39–1.98, heterogeneity *p* = 0.67, Fig. 3A). Similarly, no significant difference in the occurrence of hepatic disorder was observed between SRI patients treated with RDV and non-SRI patients treated with RDV (OR = 1.04, 95% CI = 0.43–2.53, heterogeneity *p* = 0.60, Fig. 3B).

### Mortality

**Fig. 4:**
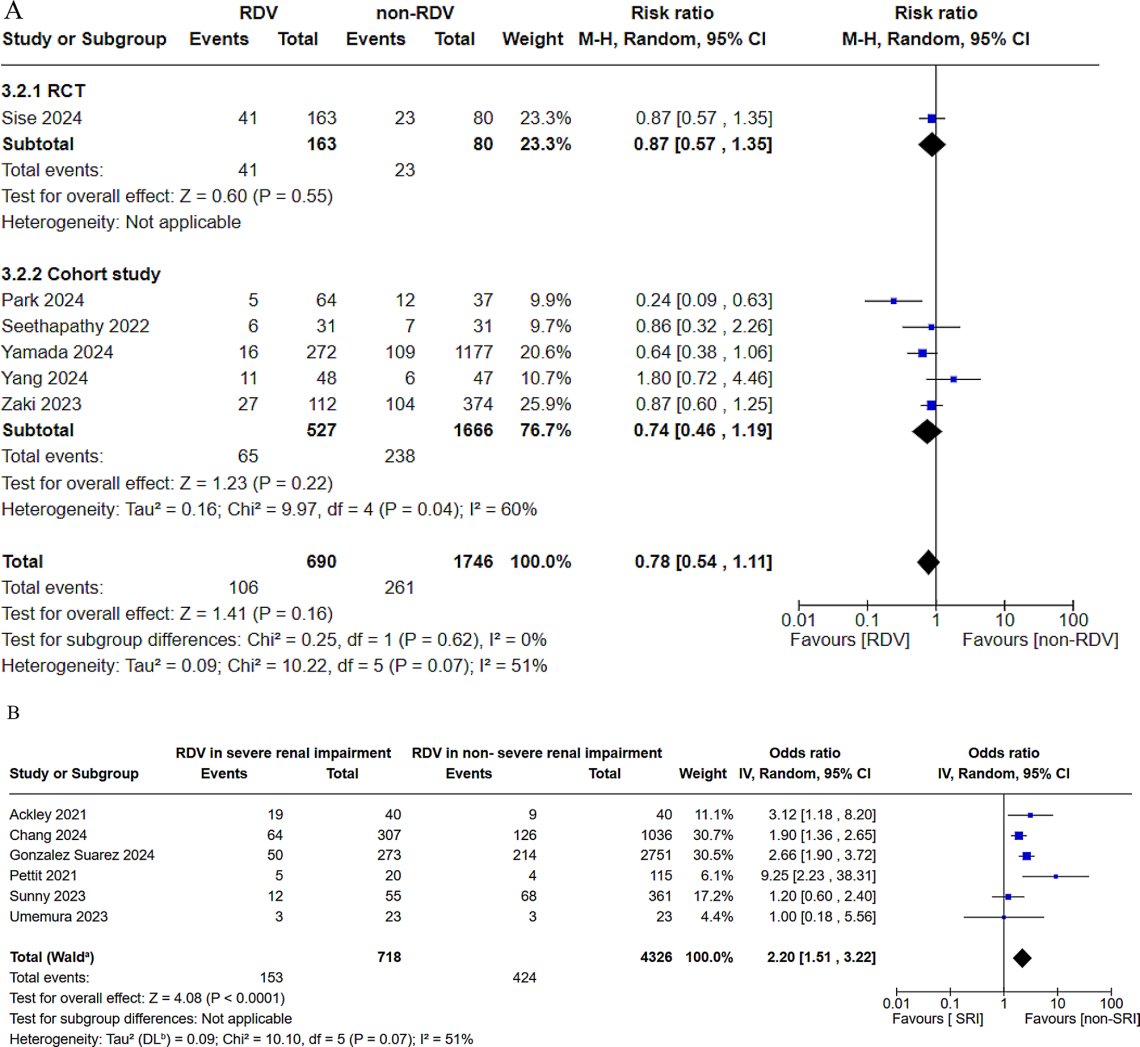
Forest plot of the risk or odds ratio for crude mortality injury between patients with severe renal impairment (SRI). This figure presents the meta-analysis of crude mortality in patients with severe renal impairment. (**A**) Patients with SRI treated with RDV vs. those not treated with RDV. (**B**) Patients with SRI treated with RDV versus patients without SRI. RDV, remdesivir; SRI, severe renal impairment; CI, confidence interval

Mortality data were extracted from 11 CSs and 1 RCT [7–9, 14, 16, 18–24] and analyzed between SRI patients treated with RDV and without RDV [7, 9, 19, 21, 22, 24] and between SRI patients treated with RDV and non-SRI patients with RDV [8, 14, 16, 18, 20, 23]. No significant difference in mortality was observed between SRI patients treated with RDV and without RDV (RR = 0.79, 95% CI = 0.55–1.15, heterogeneity *p* = 0.04, Fig. 4A). Meanwhile, SRI patients treated with RDV exhibited significantly higher mortality than non-SRI patients with RDV (OR = 2.20, 95% CI = 1.51–3.22, heterogeneity *p* = 0.07, Fig. 4B).

For mortality analyses, a sensitivity analysis was conducted, which was restricted to studies with a low ROB (Supplementary Figs. 2 and 3). In the comparison between SRI patients treated with RDV and non-SRI patients with RDV, the RR changed from 0.78 to 0.92. In the comparison between SRI patients treated with RDV and without RDV, the OR remained unchanged (2.20–2.20).

## Discussion

This meta-analysis evaluates the safety of RDV therapy in patients with COVID-19 with SRI. To the best of our knowledge, this is the first study to conduct an integrated comparison across several settings. In patients with SRI, RDV treatment reduced, rather than increased, the incidence of further renal impairment compared with that in untreated patients (Fig. 2A). SARS-CoV-2 infects host cells via the angiotensin-converting enzyme 2 (ACE2) receptor, which is predominantly expressed in alveolar epithelial type 2 cells. ACE2 expression is abundant in the proximal tubules of the kidney, and it can potentially damage proximal tubular cells [29, 30]. GS-441,524, a metabolite of RDV, is absorbed by an organic anion-transporting polypeptide [31], which may help minimize the occurrence of such disorders. RDV is formulated with the excipient sulfobutyl ether β-cyclodextrin (SBECD), a solubilizing agent that allows for its intravenous administration. The amount of SBECD present in RDV was approximately 3–6 g per 100 mg of RDV. Although this excipient is considered safe for individuals with normal renal function, its pharmacokinetics differ significantly from those of patients with renal impairment. SBECD exposure can be 21-fold higher in patients with renal dysfunction than in those with normal kidney function [6]. This increased accumulation raises concerns regarding the potential toxicity, particularly in patients with preexisting renal impairment, where SBECD clearance is likely to be diminished. Nevertheless, no significant adverse effects on clinical renal function outcomes have been reported, even in cases of renal accumulation of SBECD [32]. Furthermore, considering that the maximum recommended safe dose of SBECD is 250 mg/kg/day, with limited use over a short period (3–5 days), the benefits of RDV may outweigh the potential risks [33]. In the present study, renal function did not further deteriorate in the patients with SRI treated with RDV. By contrast, the incidence of renal impairment was higher in RDV-treated patients with SRI than in those without SRI. Additionally, the risk of death is higher in patients with renal dysfunction [34], suggesting the potential for further renal deterioration. This finding is consistent with the results of the present study (Fig. 4B), which demonstrated an increased risk of mortality in RDV-treated patients with renal dysfunction.

In the package insert, increases in AST or ALT levels were the most common adverse reactions (incidence ≥ 5%, all grades) [6]. Several studies including the GS-US-540-9015 study have shown that blood levels of GS-441,524, the active metabolite of RDV, provide valuable insights in patients with SRI [6, 35]. Pharmacokinetic studies have demonstrated that exposure to RDV and its metabolites (GS-441524 and GS-704277) is not significantly influenced by renal function or the timing of RDV administration during dialysis sessions [5]. In our meta-analysis, hepatotoxicity was generally defined as relatively severe, with ALT and AST levels exceeding five times the reference values. Therefore, elevated GS-441,524 concentrations may not significantly affect the development of hepatotoxicity.

Our meta-analysis indicated that RDV administration did not increase mortality. Other findings from this study also support the conclusion that RDV may not contribute to multi-organ failure including renal and hepatic failure leading to death. Furthermore, RDV tended to decrease mortality, although this difference was not statistically significant. However, the present study did not account for prevalent viral strains or vaccination status. Yamada et al. [7] have reported that RDV significantly reduced mortality in patients with SRI during an epidemic caused by the delta variant, for which no vaccine was available. By contrast, mortality increased among RDV-treated patients with SRI compared to those RDV-treated patients without SRI. However, these analyses (Fig. 4A and B) showed heterogeneity in the results. A cohort study has reported a significantly lower mortality rate with RDV administration in patients with SRI treated with RDV than in those not treated with RDV (Fig. 4A) [22]. Although this report may contribute to the heterogeneity of the results, based on the findings of other studies, adverse events associated with RDV would unlikely lead to increased mortality, at least if the heterogeneity were resolved. Moreover, no racial differences were observed in COVID-19 severity or mortality. However, vaccination rates, use of antibody therapy, medical service systems aimed at reducing mortality, and their availability vary among countries and even within nations during the COVID-19 pandemic. In patients with SRI treated with RDV versus those without SRI (Fig. 4B), three of the six studies were conducted in 2020, when vaccination was not yet considered widespread [14, 16, 20]. These differences may have contributed to the heterogeneity of results.

Our meta-analysis has several limitations. First, with the exception of one study, the most studies included in our meta-analysis were retrospective, and only three to six studies were analyzed in each section. Few studies adjusted for confounding related to patient backgrounds, likely due to the limited number of clinical cases included in most analyses. Our results may limit the strength of the conclusions drawn from the meta-analysis, due to the potential for bias arising from confounding factors that were not accounted for in the analysis. Second, the data regarding patient background, illness severity, SARS-CoV-2 variants, vaccination status, and antibody administration were not standardized. Third, our meta-analysis focused on renal impairment, hepatotoxicity, and mortality, which are the frequently reported adverse events. Other adverse events observed in individual studies (e.g., thrombocytopenia, anemia, and bradycardia) were excessively diverse to allow for adequate analysis. However, further studies are required to evaluate these factors.

## Conclusions

No significant difference in the safety of RDV administration was observed between patients with SRI and COVID-19 and patients without SRI or those not receiving RDV for kidney impairment, hepatic disorder, and mortality. Therefore, RDV should be actively recommended for patients with SRI.

## Electronic supplementary material

Below is the link to the electronic supplementary material.

Supplementary Material 1

## Data Availability

This manuscript has not been registered in any repository. Data will be made available on request.

## Abbreviations

ACE2: Angiotensin-Converting Enzyme 2
RDV: Remdesivir
SARS-CoV-2: Severe Acute Respiratory Syndrome Coronavirus-2.
COVID-19: Coronavirus Disease 2019
SBECD: Sulfobutyl Ether β-Cyclodextrin
SRI: Severe Renal Impairment
RCT: Randomized Controlled Trial
CSs: Retrospective And Cohort Studies
ROBINS-I V2: Risk of Bias in Non-Randomized Studies– of Interventions, Version 2
LFT: Liver Function Test
AST: Aspartate Aminotransferase
ALT: Alanine Transaminase
PRISMA: Preferred Reporting Items for Systematic Reviews and Meta-Analyses
RRs: Risk Ratios
ORs: Odds Ratios
CI: Confidence Interval
ROB: Risk Of Bias
