# Supplementary material for: Safety evaluation of remdesivir administration in patients with severe renal impairment and coronavirus disease: a systematic review and meta-analysis
Source: BMC Infect Dis. 2025 Jun 2;25:782. doi: 10.1186/s12879-025-11153-5 (PMC12128546; doi:10.1186/s12879-025-11153-5)
Supplement: Supplementary file 1 — Supplementary Material 1 [file 12879_2025_11153_MOESM1_ESM.docx]

**SUPPLEMENTAL INFORMATION**

Table S1. The risk of bias in non-randomized studies of interventions, version 2 (ROBINS-I V2) assessment

| Author, year | Bias due to confounding | Bias in classification of interventions | Bias in selection of participants into the study | Bias due to deviations from intended interventions | Bias due to missing data | Bias in measurement of the outcome | Bias in selection of the reported result | Overall risk of bias | Description |
| --- | --- | --- | --- | --- | --- | --- | --- | --- | --- |
| Ackley et al, 2021 | L | L | L | L | L | L | L | L | - |
| Schieber et al, 2021 | M | L | L | M | L | L | L | S | Bias due to confounding (M): data were not adjusted  Bias due to deviations from intended interventions (M): no presumption of effectiveness has been made. |
| Pettit et al, 2021 | M | L | L | L | L | L | L | M | Bias due to confounding (M): data were not adjusted |
| Seethapathy et al, 2022 | L | L | L | L | L | L | L | L | - |
| Koga et al, 2022 | M | S | L | L | L | L | L | S | Bias due to confounding (M): data were not adjusted  Bias in classification of interventions (S): grouping not mentioned in the method |
| Sunny S et al, 2022 | M | L | L | L | L | L | L | M | Bias due to confounding (M): data were not adjusted |
| Umemura et al, 2023 | L | L | L | L | L | L | L | L | - |
| Zaki et al, 2023 | L | L | L | L | L | L | L | L | - |
| Gonzalez et al, 2024 | M | L | L | L | L | L | L | M | Bias due to confounding (M): data were not adjusted |
| Chang et al, 2024 | M | L | L | L | S | L | L | S | Bias due to confounding (M): data were not adjusted  Bias due to missing data (S): the impact of the analysis due to missing values for some results cannot be ruled out |
| Yang et al, 2024 | L | L | L | L | L | L | L | L | - |
| Park et al, 2024 | M | L | L | L | L | L | L | M | Bias due to confounding (M): data were not adjusted |
| Yamada et al, 2024 | M | L | L | L | L | L | L | M | Bias due to confounding (M): data were not adjusted |

L, low; M, moderate; S, serious


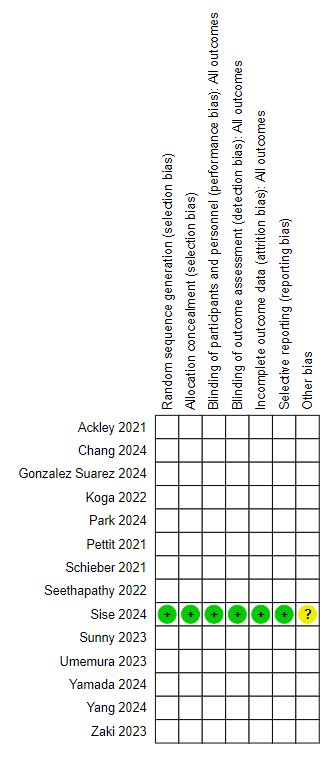

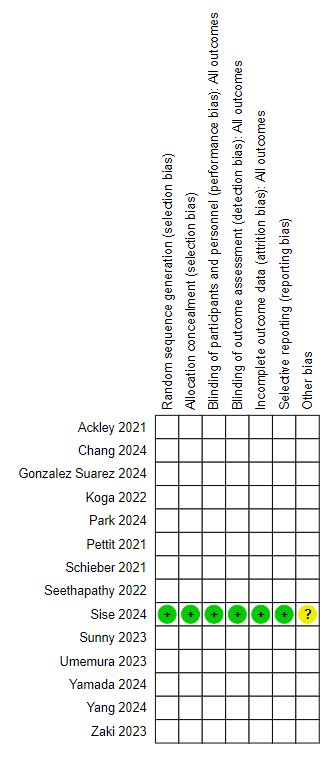

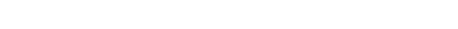

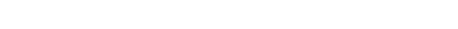


Fig. S1 Risk of bias for included randomized control trial


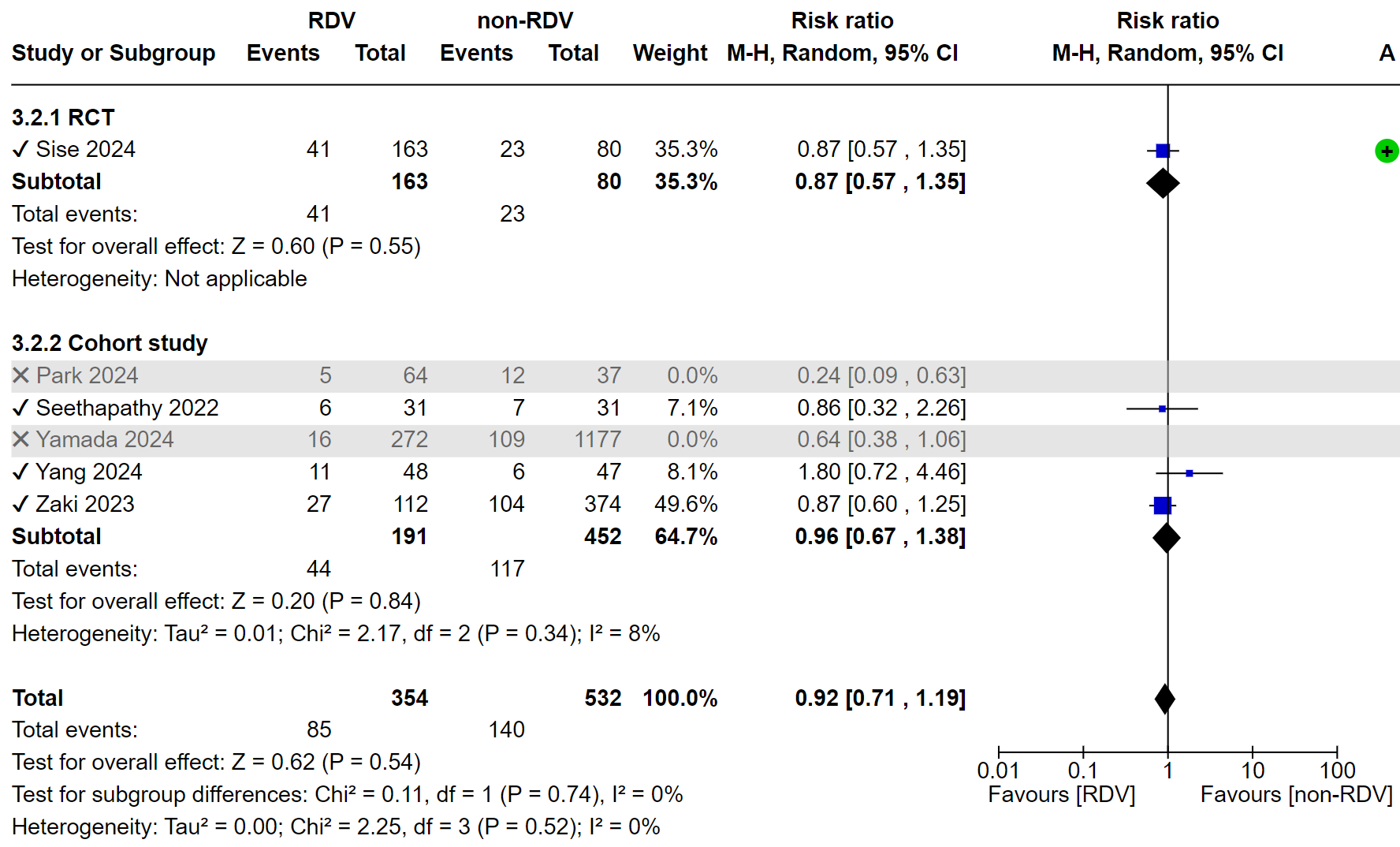
 Fig. S2 Forest plot for sensitivity analysis restricted to low risk of bias studies (patients with severe renal impairment treated with RDV versus those not treated with RDV).

The x mark was excluded in the sensitivity analysis and reanalyzed because the risk of bias assessment was moderate or serious.


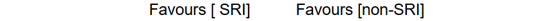

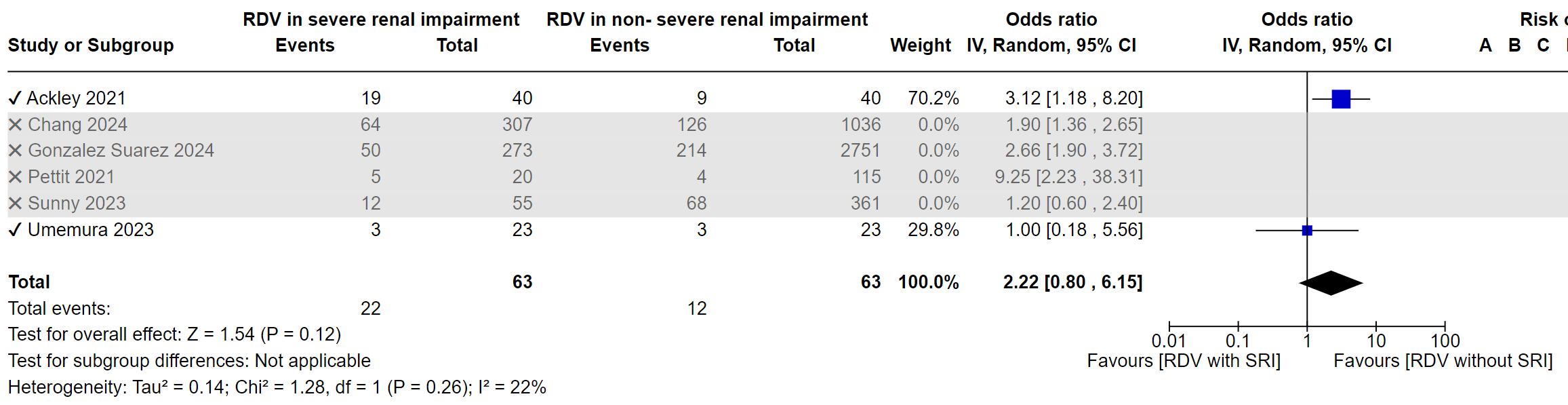


Fig. S3 Forest plot for sensitivity analysis restricted to low risk of bias studies (patients with severe renal impairment (SRI) treated with RDV versus non-SRI patients treated with RDV).

The x mark was excluded in the sensitivity analysis and reanalyzed because the risk of bias assessment was moderate or serious.
